# SARS-CoV-2 viral dynamics of the first 1000 sequences from Vietnam and neighbouring ASEAN countries

**DOI:** 10.1016/j.ijregi.2022.01.008

**Published:** 2022-01-20

**Authors:** Nghiem Xuan Hoan, Srinivas Reddy Pallerla, Pham Xuan Huy, Hannah Krämer, Truong Nhat My, Tran Thanh Tung, Phan Quoc Hoan, Nguyen Linh Toan, Le Huu Song, Thirumalaisamy P. Velavan

**Affiliations:** 1 Department of Molecular Biology, 108 Military Central Hospital, Hanoi, Vietnam; 2 Vietnamese-German Center for Medical Research (VG-CARE), Hanoi, Vietnam; 3 Institute of Tropical Medicine, University of Tübingen, Tübingen, Germany; 4 Vietnamese Military Medical University, Hanoi, Vietnam

**Keywords:** SARS-CoV-2, COVID-19, Vietnam, ASEAN, variants of concern, delta variant, B.1.614.2, VOC

## Abstract

**Objective:**

The regional distribution and transmissibility of existing COVID-19 variants of concern (VOC) has led to concerns about increasing transmission, given the ability of VOCs to evade immunity as breakthrough infections become more prevalent.

**Methods:**

SARS-CoV-2 genomes (*n* = 277) were sequenced and analysed alongside all available genomes from Vietnam and ASEAN countries to understand the phylodynamics. The observed lineages were assigned using Pangolin nomenclature, and spread patterns were investigated.

**Results:**

Between January and November 8, 2021, VOCs, including alpha (B.1.1.7), beta (B.1.351), gamma (P.1), and delta (B.1.617.2), were observed across the ASEAN countries. While alpha and delta were the major VOCs in nine ASEAN countries, delta was predominant. The alpha VOC was first reported by Singapore, beta by Malaysia, gamma by the Philippines, and delta by Singapore. Of the first 1000 genomes analysed from Vietnam, alpha and delta were the most represented, with delta being the dominant VOC from May 2021. The delta variant was introduced in early January 2021, and formed a large cluster within the representative genomes.

**Conclusion:**

Spatial and temporal monitoring of SARS-CoV-2 variants is critical to the understanding of viral phylodynamics, and will provide useful guidance to policy makers for infection prevention and control.

## Highlights

• The first 1000 SARS-CoV-2 genome sequences from Vietnam were analyzed.
• Delta was the major VOC in nine ASEAN countries.
• There are limited sequencing capacities in Laos, Brunei, and Myanmar.
• Strong epidemiological patterns of virus spread were evident after the introduction of VOCs in ASEAN.

## Introduction

Given the ongoing COVID-19 pandemic, and the need to be prepared for the threat of emerging variants, immediate and collective action is needed. Variants of concern (VOCs) appear to spread more easily and rapidly than other variants, which may lead to more COVID-19 cases (Manouana et al., 2021). Although available COVID-19 vaccines protect against severe forms of COVID-19, the effectiveness has been diminished by breakthrough infections with the delta variant (Levine-Tiefenbrun et al., 2021), which at the time of writing was the dominant strain of SARS-CoV-2 worldwide (WHO, 2021a).

COVID-19 cases caused by the alpha variant (B.1.1.7) have been reported in 196 countries, the beta variant (B.1.351) in 145 countries, the gamma variant (B.1.1.28) in 99 countries, and the delta variant (B.1.617.2) in 193 countries (WHO, 2021a). Vietnam, a low–middle-income country (LMIC) in Southeast Asia, has experienced three waves of cluster transmission (January to April 2020; July and August 2020, December 2020 to March 2021). Despite the difficulties many countries have faced in controlling transmission, as of April 2021, Vietnam had controlled these successfully. Since then, the country has been experiencing an exponential increase in COVID-19 cases (from 1537 cases and 35 deaths as of January 18, 2021 to 968684 cases with 22531 deaths by November 8, 2021) despite previously successful public health measures (Song et al., 2021; WHO, 2021b). It was this observation, among others, that prompted us to build up Vietnam's sequencing capacity.

COVID-19 vaccination in Vietnam began on March 8, 2021, with eight vaccines approved for use in the country as of January 5, 2022. These include two based on RNA (Moderna's mRNA-1273 and Pfizer/BioNTech's BNT162b2), three based on non-replicating viral vectors (Gamaleya's Sputnik V, Johnson & Johnson's Ad26.COV2.S, and AstraZeneca's AZD1222), two inactivated vaccines (Bharath Biotech's Covaxin and Sinopharm's BBIBP-CorV), and one protein subunit vaccine (Abdala CIGB-66). Lack of knowledge about the regional distribution and transmissibility of existing and emerging VOCs will have significant implications for infection prevention and control measures. The rapid temporal and spatial evolution of SARS-CoV-2 is not yet fully understood, but the increasing availability of gene sequence data is helping the study of viral transmission dynamics. Viral genomes are an independent source of information on epidemiological processes, which can be used to support and confirm epidemiological findings obtained through routine surveillance. Therefore, it is essential to monitor whether variants are spreading more readily, as evidenced from several countries where VOCs have been associated with an increased risk to public health (WHO, 2021a).

In order to understand the origin of SARS-CoV-2, the dynamics of transmission, different patterns of spread, and its evolution from an epidemiological perspective, SARS-CoV-2 genomes from Vietnam were sequenced at the Vietnamese-German Center for Medical Research and analyzed alongside genomes from different ASEAN countries deposited on GISAID.

## Methods

### SARS-CoV-2 viral phylodynamics

All SARS-CoV-2 genomes from ASEAN countries that were deposited on GISAID up to November 8, 2021 were retrieved (Shu and McCauley, 2017). These included sequences from Laos (*n* = 18), Brunei (*n* = 38), Myanmar (*n* = 90), Vietnam (*n* = 1051), Cambodia (*n* = 1468), Malaysia (*n* = 4795), Thailand (*n* = 6497), Indonesia (*n* = 8578), Singapore (*n* = 9385), and Philippines (*n* = 12739) (Table 1). Of those from Vietnam, 277 SARS-CoV-2 genomes were sequenced in our laboratory (up to November 8, 2021) using the Oxford Nanopore NGS sequencing methodology, as described earlier (Manouana et al., 2021). The genome sequences were aligned using a multiple sequence alignment (MAFTT) algorithm (Katoh et al., 2002), with hCoV-19/Wuhan/WIV04/2019 (MN996528.1) as a reference genome. The lineages were obtained using the Pangolin tool (Rambaut et al., 2020) Subsequently, using the aligned sequences, a phylogenetic tree was reconstructed to show the different lineages, using the Nextstrain algorithm, as implemented in the web tool (Hadfield et al., 2018).

**Table 1:** SARS-CoV-2 genomes from ASEAN countries that were deposited in GISAID, and the lineages circulated up to October 2021.

ASEAN Countries	GISAID (*n*)	Earliest sampled sequence	First sequence deposited	Tracked lineages (%)	First reported VOCs
Laos	18	04.11.2020	08.20.2021	Not available	Not available
Brunei	38	03.11.2020	05.05.2020	B.1.617.2 (91); B.1.1 (6); B.1.351 (3)	01.20.2021 (beta) 07.21.2021 (delta)
Myanmar	90	04.22.2020	08.12.2020	B.1.36.16 (43); B.1.617.2 (40); Others (27)	05.28.2021 (alpha) 05.28.2021 (delta)
Vietnam	1051	01.22.2020	02.10.2020	B.1.617.2 (80); B.1.1 (7); Others (6); B.1 (4)	12.15.2020 (alpha) 04.21.2021 (delta)
Cambodia	1468	01.27.2020	02.21.2020	B.1.1.7 (57); B.1.617.2 (36); Others (3); A.23.1 (1); B.1.36.16 (1); Q.1 (1); B.1 (1)	02.03.2021 (alpha) 05.25.2021 (delta)
Malaysia	4795	01.24.2020	02.14.2020	B.1.617.2 (58); B.1.524 (11); AU.2 (9); B.1.351 (6); Others (3); B.1.466.2 (2); B.1 (2); B.1.36.16 (2); B.1.459 (2); B.6 (1); B.1.470 (1); B.6.1 (1); B.1.1.7 (1); B.1.1.526 (1); B (1)	12.02.2020 (beta) 12.28.2020 (alpha) 02.09.2021 (delta)
Thailand	6497	01.08.2020	17.01.2020	B.1.1.7 (35); B.1.617.2 (45); B.1.36.16 (7); A.6 (3); Others (3); B.1 (2); B.1.351 (2); B.1.1 (1); B.1.524 (1)	12.21.2020 (alpha) 01.09.2021 (delta) 02.03.2021 (beta) 04.05.2021 (gamma)
Indonesia	8578	03.01.2020	03.04.2020	B.1.617.2 (54); B.1.466.2 (22); B.1.470 (7); Others (3); B.1(3); B.1.1.398(2); B.1.459(2); B.1.1(2); B.1.468(1); B.1.1.7(1); B.1.36.19(1); B (1)	01.05.2021 (alpha) 01.25.2021 (beta) 07.29.2021 (delta)
Singapore	9385	01.23.2020	02.03.2020	B.1.617.2 (73); B.6.6 (9); Others (3); B.1.1.7(2); B (1); B.1.1(1); B.1 (1); B.1.351.3(1); B.1.351 (1); B.1.524 (1); B.1.466.2 (1); B.1.617.1 (1)	12.08.2020 (alpha) 02.06.2021 (delta) 02.07.2021 (beta) 04.04.2021 (gamma)
Philippines	12739	01.23.2020	02.06.2020	B.1.351 (25); B.1.617.2 (23); B.1.1.7 (21); B.1.1.63 (8); P.3 (4); B.1.1 (4) Others (4); B.1.1.28 (3); B.1 (2); B.6 (1)	12.10.2020 (alpha) 01.31.2021 (beta) 02.21.2021 (gamma) 04.09.2021 (delta)

## Results

As of November 8, 2021, a total of 44659 SARS-CoV-2 genomes from ASEAN countries had been deposited on GISAID (Table 1), the largest number from the Philippines (*n* = 12739). All circulating VOCs, including alpha (B.1.1.7), beta (B.1.351), gamma (P.1), and delta (B.1.617.2), were observed in all ASEAN countries. Delta was the predominant VOC among the analyzed sequences for nine ASEAN countries (91% in Brunei to 23% in the Philippines), followed by alpha VOC. Beta VOC was the third most reported in six ASEAN countries (Philippines 25%, Malaysia 6%, Brunei 3%, Thailand 2%, Singapore 1%, and Indonesia 0.26%). Gamma VOC was reported in three countries (8, 4, and 1 sequence from Singapore, Philippines, and Thailand, respectively; each < 1%) (Table 1). Only partial genomes for SARS-CoV-2 were deposited from Laos, and no lineages were described. Among the ASEAN countries, alpha VOC was first reported by Singapore, beta VOC by Malaysia, gamma VOC by the Philippines, and delta VOC by Singapore (Table 1).

**Figure 1:**
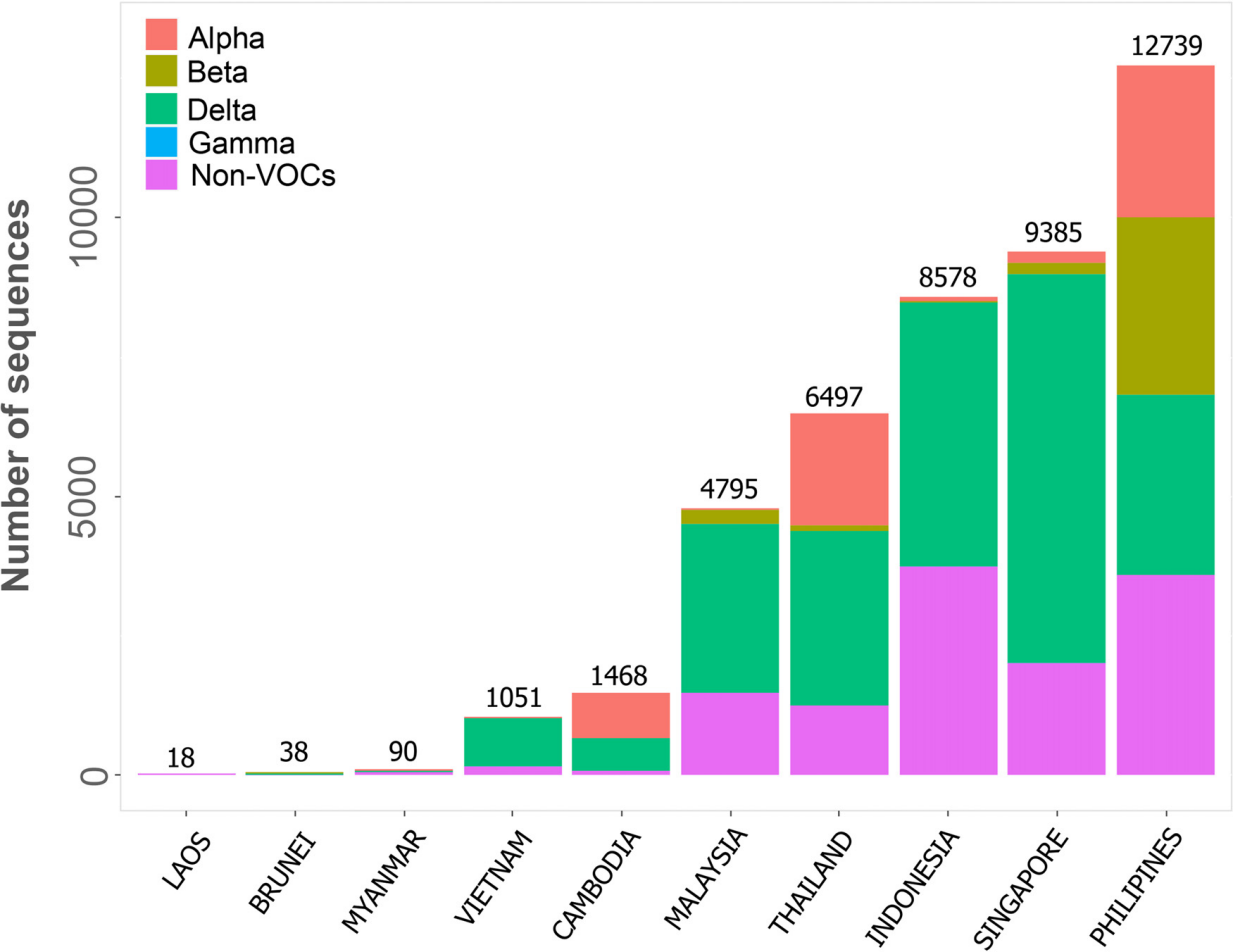
Distribution of SARS-CoV-2 variants of concern in ASEAN countries between December 2020 and November 8, 2021.

The distribution of SARS-CoV-2 VOCs in ASEAN countries between December 2020 and November 8, 2021, based on available sequences and lineages, is shown in Figure 1; alpha and delta were the largest contributors. Before the introduction of VOCs, non-VOCs were equally represented in these countries. In the Philippines, an even distribution of alpha, beta, gamma, and delta VOCs was observed across all analysed sequences. However, from early 2021, delta became the predominant strain in the Philippines, as well as in Singapore, Indonesia, Vietnam, Thailand, and Malaysia (Figure 1).

**Figure 2:**
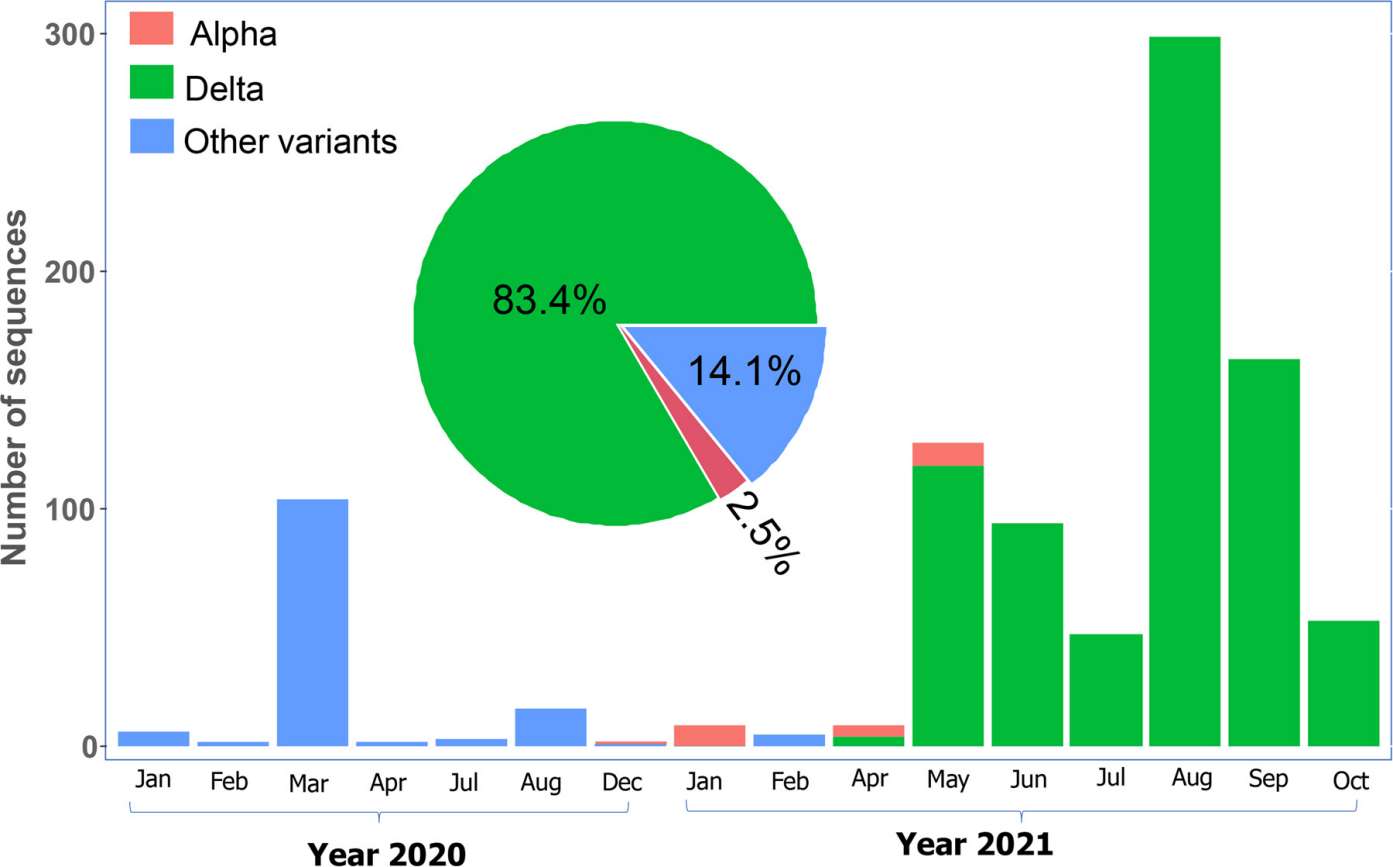
Distribution of SARS-CoV-2 lineages and VOCs in Vietnam between January 2020 and November 8, 2021.

Based on the available sequences, from December 15, 2020 to May 22, 2021, only the alpha variant was in circulation in Vietnam. The delta variant was first detected in the population on April 21, 2021, and soon became prevalent, accounting for most reported cases. Across all analyzed sequences, the delta and alpha variants accounted for 83% and 3%, respectively, with the remaining 14% associated with other viral lineages, as observed in GSAID (Figure 2).

**Figure 3:**
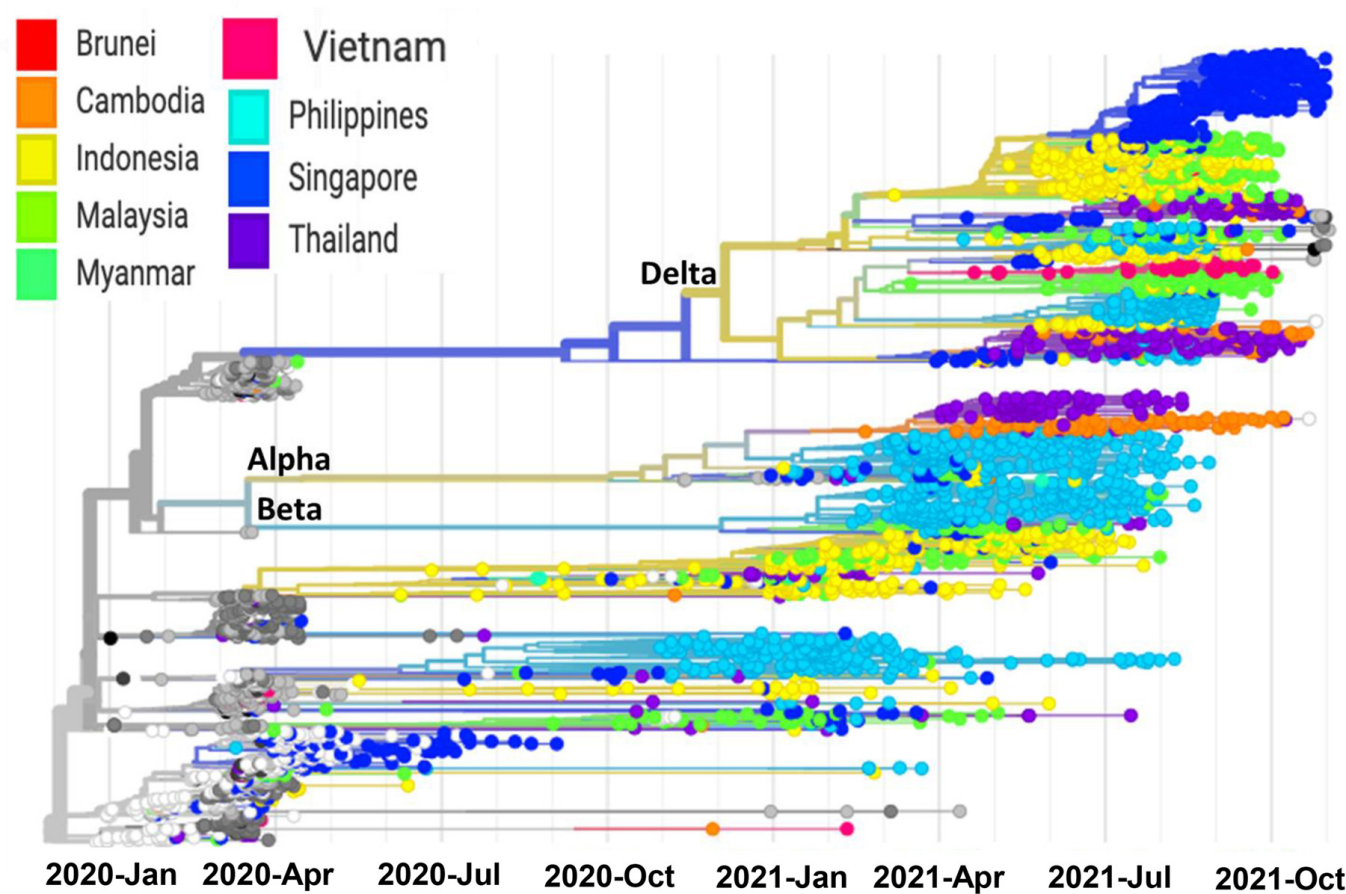
Phylogenetic tree representing SARS-CoV-2 variants over space and time from nine ASEAN countries represented by different colours, reconstructed using Nextstrain algorithms (https://nextstrain.org/).

The phylogenetic tree in Figure 3 shows the SARS-CoV-2 variants circulating in nine ASEAN countries. The alpha and delta variants appeared from December 2020, from two independent clades, and became present in all ASEAN countries except Brunei, where alpha was not reported. The predominant delta variant, arising in ASEAN countries in early January 2021, formed a large cluster, which included representative genomes from all ASEAN countries (Figure 3). Our phylogeographic reconstruction of past virus spread patterns suggested a strong epidemiological link between ASEAN countries, and indicated a changing pattern of virus spread after the introduction of VOCs across the region in early 2021. In almost all cases, the initial spread of SARS-CoV-2 VOCs into individual ASEAN countries seemed to originate from countries outside ASEAN.

## Discussion

Among other factors, the lack of genomic data on circulating SARS-CoV-2 variants will further limit the capacity to control the pandemic in ASEAN countries, where some countries have limited or no resources in terms of testing and/or sequencing capacity, as observed in Laos, Brunei, and Myanmar. Recently, the rapid deployment of SARS-CoV-2 sequencing by several laboratories in Vietnam has enabled the generation and sharing of genomic data. For example, of the 1051 SARS-CoV-2 sequences that had been submitted from Vietnam by November 8, 2021, 546 were deposited in the final 40 days (October 1 to November 8). Sequencing and genomic surveillance efforts relating to various VOCs and variants of interest have undoubtedly increased in the ASEAN region, in part due to global interest in understanding viral spread. However, data from Laos, Brunei, and Myanmar are still lacking. Nevertheless, the available data demonstrate that SARS-CoV-2 variants have emerged and spread rapidly within the ASEAN countries. The fact that the availability of SARS-CoV-2 sequences only increased to a considerable level within recent months limits the interpretation of variant spread in the earlier stages of the pandemic.

Vietnam has responded comprehensively and quickly to the pandemic. From the beginning of the outbreak, the government effectively engaged state institutions in response efforts. There was an immediate directive to produce guidelines for hygiene measures, and a four-tier quarantine structure was implemented. Strong restriction measures on movements via road links between neighbouring countries, as well as a limited air travel, may have also held up the spread of virus variants. High rates of COVID-19 testing in Vietnam (764044 tests per 1 million inhabitants) have led to the early identification of cases. However, Vietnam has been experiencing an exponential increase in COVID-19 cases since April 2021. Whereas the earlier outbreaks in Vietnam were caused by imported cases (forming a cluster of cases up to April 2021) (WHO, 2021a), the country now has to deal with community transmissions, which may lead to more severe waves in the future.

It has been observed that there is a strong epidemiological link between ASEAN countries and the changing patterns of virus spread after the introduction of VOCs in the region. However, the origins of introduced VOCs remain unclear because of the low proportion of sequenced viruses across the region. The available sequencing data contain little evidence of positive selection in non-synonymous regions and only possible evidence of phylogenetic expansion of viral lineages. These factors can only be well understood if large, representative samples across time and space become available to provide valuable evidence of viral movements between countries.

All ASEAN countries have implemented the COVID-19 vaccination program. While Vietnam has a fully vaccinated rate of 57%, Brunei and Singapore have achieved the highest coverage (both 87%) and Myanmar the lowest (28%), as of January 5, 2022. Although extensive introduction of COVID-19 vaccines has been documented in ASEAN countries, with the advent of the omicron VOC, countries should also accelerate their sequencing capacities so that important gaps in knowledge about VOCs, particularly with regard to altered responses to vaccines, treatments, and transmissibility, can be addressed.

This study provides comprehensive insights into the temporal and spatial occurrence of the tracked lineages in Vietnam and in neighbouring ASEAN countries.

## Author contributions

LHS, TPV, and NLT designed the study and contributed materials. HK, TNM, TTT, and PXH performed NGS sequencing. PQH contributed to the diagnoses of COVID-19. Data were analyzed by NXH, TPV, and SRP. TPV and NXH wrote the manuscript and HK revised the draft.

## Declaration of Competing Interests

All authors report no competing interests.
